# Derivation of Stem Cell-like Cells From Spherical Culture of Astrocytes for Enhanced Neural Repair After Middle Cerebral Artery Occlusion

**DOI:** 10.3389/fbioe.2022.875514

**Published:** 2022-04-04

**Authors:** Dan Zhu, Zheming Cao, Xiaoyang Pang, Wei Jiang, Chihao Li, Xing Zhang, Xibin Tian, Haijun Tu, Panfeng Wu, Hemin Nie

**Affiliations:** ^1^ Department of Biomedical Sciences, College of Biology, Hunan University, Changsha, China; ^2^ Department of Orthopedics, Xiangya Hospital Central South University, Changsha, China; ^3^ Department of Pharmaceutics, College of Biology, Hunan University, Changsha, China

**Keywords:** astrocyte, neural stem cell, cell transplantation, neural differentiation, tissue regeneration

## Abstract

Neural precursor cells (NPCs) tend to aggregate and develop into three-dimensional (3D) spheres, which in turn help maintain the stemness of the cells. This close relationship between spherical environments and cell stemness direct us to assume that 3D spheres of astrocytes (ASTs) may facilitate the acquisition of stem cell-like features and generate sufficient seed cells for the regeneration of neurons. *In vitro* results confirmed that mouse ASTs cultured on agarose surfaces spontaneously formed cell spheres and exhibited molecular features similar to stem cells, particularly capable of further differentiating into neurons and forming functional synaptic networks with synchronous burst activities. RNA-sequencing results revealed the similarity between AST-derived stem cells (A-iSCs) and NPCs in global gene expression profiles. The potency of A-iSCs in repairing neural injuries was evaluated in a mouse model of middle cerebral artery occlusion. It was observed that the transplanted A-iSCs expressed a series of markers related to neural differentiation, such as NeuN, Tuj1, and Map2, indicating the conversion of the transplanted A-iSCs into neurons in the scenario. We also found that the injured mice injected with A-iSCs exhibited significant improvements in sensorimotor functions after 8 weeks compared with the sham and control mice. Taken together, mouse ASTs form cell spheres on agarose surfaces and acquire stem cell-associated features; meanwhile, the derived A-iSCs possess the capacity to differentiate into neurons and facilitate the regeneration of damaged nerves.

## 1 Introduction

Stroke is globally one of the main causes leading to deaths and disabilities. Thrombolytic treatments are effective, but they can only cover less than 10% of patients with stroke as the therapeutic time window for this type of treatment is extremely narrow (4.5 h after the onset of stroke) (Auriel and Bornstein, 2010). The resident neural precursor cells (NPCs) contribute to ongoing postnatal neurogenesis. In animal models of stroke, i.e., middle cerebral artery occlusion (MCAO), neurological behavioral deficits have been remarkably improved through the transplantation of NPCs (Mack, 2011). A series of engineering strategies have been developed to advance cell therapy treatments. However, the lack of active progenitor cells has restricted the regenerative potential of these cell therapy strategies for neural injuries. Therefore, the development of approaches for obtaining sufficient active progenitor cells is highly demanded in the hope of functional reconstruction of damaged nerve tissues.

Neurons cannot be replenished after damage in the vast majority of the mammalian central nervous system (CNS). As a result, neuronal loss is considered definitive in neurological diseases such as stroke. A series of studies have suggested that NPCs resembling glial cells solely reside in the subgranular zone of the hippocampus and the ventricular subependymal zone. In this sense, the absence or low supply of NPCs in the CNS may have contributed to the limited regeneration of neural injuries in the brain. Astrocytes are glial cells located in all regions of the brain and the most plentiful cells in the human brain (Molofsky et al., 2012; Verkhratsky et al., 2012), which have long been simply considered as supporting components in neural tissues (Sofroniew and Vinters, 2010). However, with increasing evidence, a variety of essential functions have been established for astrocytes in neural development (Eroglu and Barres, 2010; Verkhratsky, et al., 2012). The ready availability and their features such as proliferation and proximity in lineage to neurons make astrocytes an outstanding candidate cell source for NPCs (Amamoto and Arlotta, 2014).

Terminally differentiated somatic cells are capable of being converted into pluripotent stem cells through the forced up-expression of a specific set of transcription factors (Ladewig et al., 2013). However, the involvement of ectopic transgenes bottlenecks their practical application. Alternatively, great attention has been paid to other inducers for cell reprogramming, such as proteins, RNAs (Warren et al., 2010), microRNAs (Anokye-Danso et al., 2011), and defined chemicals (Zhu et al., 2010). In addition, it was found that the cell microenvironment could induce a variety of behavioral changes including proliferation, migration, apoptosis, and differentiation, suggesting that cell reprogramming might be achieved by manipulating the cell microenvironment (Eshghi and Schaffer, 2008). Compared to conventional two-dimensional (2D) cultivation, cells in three-dimensional (3D) environments demonstrate differentiated cell morphology, cell–cell contact, and cell–matrix interactions (Nelson and Bissell, 2006). A series of 3D cell culture systems, such as porous scaffolds and hydrogels, have been developed to facilitate 3D cellular spheres. Recently, self-organized 3D cellular spheres emerge as another 3D cell culture approach by taking advantage of the natural aggregation tendency of some specific cell types, without involving 3D scaffolding materials. Previous studies have suggested the linkage between cellular spheres and cell stemness (Su et al., 2013a; Su et al., 2013b; Caiazzo et al., 2016). This close linkage between 3D cellular spheres and stem cell characteristics prompts us to assume that forming astrocyte spheres may facilitate the acquiring of stem cell-like features and therefore generate sufficient amount of NPC-like cells.

Herein this work, we prepared low-attachment surfaces by coating regular cell culture dishes with agarose and generated 3D spheres of mouse astrocytes through spontaneous aggregations of the cells on agarose surfaces. Astrocytes in spheres showed an upregulation of NPC markers compared with the cells in 2D environments. Subsequently, we transplanted the astrocyte-induced stem cells (A-iSCs) as seed cells into damage sites in MCAO model mice to verify the therapeutic effects of the cells *in vivo*. Histological and physiological analyses revealed that the transplantation of A-iSCs promoted the functional recovery of MCAO mice.

## 2 Materials and Methods

### 2.1 Isolation and Primary Culture of Astrocytes, P56-NPCs and P0-NPCs

Primary astrocytes were isolated from the cerebra of postnatal C57BL/6 mouse from Day 1 to Day 2. After removing the meninges, the cerebral cortex tissues were trypsinized and mechanically disrupted to obtain a single-cell suspension. Cells were centrifugated for 5 min (1,000 rpm), re-dissociated, and cultivated in DMEM, and then supplemented with 10% fetal bovine serum for 24 h. The culture medium was then changed and replenished every 3 days later. When reaching a confluence of 80%, the monolayer cells were passaged through trypsinization after removing loosely-attached cells. As positive controls in RNA-sequencing, NPCs at two different stages were harvested from mouse brains. P56-NPCs were obtained from C57BL/6J adult mice (8 weeks) exactly following the protocol (Ortega et al., 2011). The subependymal zone (SEZ) is carefully dissected from the brain of adult mice and enzymatically dissociated to obtain the cell suspension.

After passing through a 70 μm cell strainer and three rounds of centrifugation at 200 *g* for 5 min, 450 *g* for 10 min, and 250 *g* for 7 min at 4°C, the cells were resuspended in a pre-warmed culture medium supplemented with B27. Usually, 30,000–40,000 P56-NPCs were obtained from each mouse brain. In parallel, P0-NPCs were obtained from the C57BL/6J mice following a revised protocol based on literatures (Lee et al., 2017; Jiang et al., 2019). Briefly, the hippocampus was dissected from postnatal Day-0 wild-type C57BL/6J mice in a tissue culture hood and placed into a 15 ml tube with dissection solution on ice. Hippocampal preparation was washed and dissociated through trypsinization and resuspension to obtain single-cell suspensions, which were then centrifugated at 800 *g* for 5 min to obtain P0-NPCs.

### 2.2 Preparation of Agarose-Coated Dishes

2X concentrate DMEM medium was prepared by dissolving 26.8 g of DMEM high-glucose powder (GIBCO) and 7.4 g of sodium bicarbonate in 1,000 ml of deionized water (Su et al., 2013b). After passing through a 0.22 μm filter, the medium was pre-warmed to 37°C. In parallel, 1% agarose solution was prepared by dissolving agarose G-10 (BIOWEST) in deionized water and heating until boiled. An equal volume of pre-warmed 2X concentrate DMEM medium was mixed with 1% agarose solution, which was then filled in regular 60-mm cell culture dishes. Dishes with an around 4-mm-thick layer containing 0.5% of gel would be prepared after incubating at room temperature. ASTs were loaded onto the agarose-coated dishes (6 × 10^6^ cells per dish) and cultured at 37°C with 5% of CO_2_ to induce the formation of AST spheres. The cell culture medium was renewed every 2 days.

### 2.3 Immunocytochemistry

The cells derived on agarose dishes were transferred onto regular cell culture dishes and cultured in an expansion or differentiation medium (Stem Cell Inc.). After being fixed with 4% of paraformaldehyde and permeabilized with 0.2% of TritonX-100, the cells were incubated with PBS containing 5% of milk and 2% of normal goat serum (Su et al., 2013b). Then the cells were incubated with primary antibodies, including Tuj1 (1:1,000, Abcam), GFAP (1:500, Millipore), Map2 (1:1,000, Abcam), Oct4 (1:1,000, Abcam), Sox2 (1:50, Life Technologies), DDC ((1:100, Novus Biologicals), NeuN (1:50, Millipore), and DAPI (1:1,000, Sigma). Secondary antibodies included Alexa Fluor 488 donkey anti-rabbit IgG (1:1,000, Life Technologies) and Alexa Fluor 546 donkey anti-mouse IgG (1:1,000, Life Technologies). The nuclei of the cells were stained with Hoechst 33342 (Life Technologies).

### 2.4 Flow Cytometry

The cells derived on agarose dishes were transferred onto regular cell culture dishes and cultured in an expansion or differentiation medium (Stem Cell Inc.). The cells were trypsinized and suspended at 1 × 10^6^ cells/ml in DMEM medium containing 3% of BSA, which were then incubated at 37°C for 30 min, either alone or in the presence of primary antibodies for 1 h, and then they were resuspended in 100 ml of 488-labeled secondary antibodies (1:1,000, Invitrogen). Primary antibodies included Sox2 (1:50, Abcam), Map2 (1:50, Abcam), and Tuj1 (1:50, Abcam). At the end of the treatment, cells were centrifugated and resuspended in ice-cold PBS for a flow cytometry analysis. A side population analysis was carried out through the FACSVantage SE software (BD Biosciences).

### 2.5 Western Blotting

The total protein of cells was extracted through RIPA buffer containing a protease inhibitor cocktail (Roche), which was separated on an 8% gradient SDS-PAGE and transferred onto a PVDF membrane (Millipore). The membrane was incubated with the primary antibodies of interest, and HRP-conjugated secondary antibodies against mice or rabbits. Primary antibodies of interest included Nanog (1:1,000, Abcam), Sox2 (1:200, Life Technologies), Oct4 (1:1,000, Abcam), beta-Actin (1:5,000, Santa Cruz), BDNF (1:1,000, Abcam), and NGF (1:1,000, Abcam). Secondary antibodies conjugated with HRP included Goat-Anti Mouse IgG (1:5,000, SouthernBiotech), and Goat-Anti Rabbit IgG (1:2000 Thermo Scientific).

### 2.6 Alkaline Phosphatase (ALP) Staining

The cells were fixed in 60% acetone prediluted in 1.5 M of sodium citrate solution for 60 s, which were washed with PBS and then stained with BCIP/NBT solution (Sigma) according to the manufacturer’s manual.

### 2.7 Gene Expression Profiling and Bioinformatic Analyses

Total RNA was isolated from mouse astrocytes (negative control), mouse brain-derived NPCs (positive controls), and A-iSCs through Trizol. A total amount of 3 μg of RNA per sample was collected for sequencing. The sequencing libraries were generated through a NEBNext^®^ UltraTM RNA library prep kit (Xie et al., 2020). The clustering of the index-coded samples was performed on a cBot Cluster Generation System through a TruSeq PE cluster kit v3-cBot-HS (Illumia) according to the protocols. After cluster generation, the library preparations were sequenced on a platform produced by Illumina Hiseq, where 125 bp/150 bp paired-end reads were generated. Clean reads were obtained through removing reads containing adapters and ploy-N as well as low-quality reads. All the downstream analyses were based on high-quality clean data. Differential expression analysis was performed on two groups through the DESeq2 R package (1.16.1). Genes with an adjusted *p*-value < 0.05 found by DESeq2 were assigned as differentially expressed. Gene ontology (GO) enrichment and KEGG pathway analyses of differentially expressed genes were implemented through the clusterProfiler R package (Xie, et al., 2020).

### 2.8 *In Vitro* Differentiation of A-iSCs

A-iSCs were plated in 24-well plates precoated with poly- *l* -ornithine (Sigma). For neuron and astrocyte differentiation, the cells were cultured for 2 weeks in DMEM-F12 medium (1:1) with 2% of the B27 supplement.

### 2.9 Calcium Imaging

The cells were incubated in a culture medium supplemented with 5 μM of Fluo-8 AM (AAT Bioquest) for 30 min at 37°C. The cells were excited at 490 nm and the emission at 525 nm was monitored through a Nikon Super Fluor objective, a Hamamatsu ORCA-ER digital camera (Hamamatsu), and a Sutter DG5 optic switcher (Sutter Instrument). Imaging data were acquired and analyzed through Simple-PCI software (Hamamatsu).

### 2.10 Patch Clamp Analysis

The cells cultured on glass coverslips were transferred to a chamber that was mounted to an x-y stage and was continuously superfused with artificial cerebrospinal fluid at 2 ml/min (Su et al., 2013b). The cells were visualized at room temperature through an upright near-infrared differential interference contrast microscope with an objective of water immersion. A single-cell current clamp was carried out with a 700A multi-clamp amplifier (molecular devices). The patch pipette (tip resistance 5–7 MΩ) contained 120 mM potassium gluconate, 20 mM KCl, 10 mM NaCl, 10 mM EGTA, 1 mM CaCl_2_, 4 mM Mg ATP, 0.4 mM Na GTP, and 10 mM HEPES/KOH (pH 7.2, 290 mmmol/kg).

### 2.11 Generation of MCAO Model and A-iSCs Transplantation

All animal experiments were performed with approval from Hunan University Animal Ethics Committee (No. SYXK [Xiang] 2018-0006). Briefly, adult male C57BL/6 mice were housed with access to food and water ad libitum within a 12 h light/dark cycle. After being anesthetized with 1% of ketamine (30 mg/kg, i.p.) and xylazine hydrochloride (4 mg/kg, i.p.), the mice were ready for surgical procedures, whose body temperature was maintained at 37°C through a rectal probe and a heating pad during the entire surgery and recovery.

A method reported by Longa was followed to establish model mice with MCAO (Longa et al., 1989). Specifically, a blunt-ended monofilament (4-0, Ethicon) was inserted into the internal carotid artery to occlude the middle cerebral artery, which was carefully removed 1.5 h after the occlusion. 24 h later, the acute neurological severity of the mice was checked through circling behavior and forelimb/hindlimb placement tests, and appropriate mice with stroke were chosen for animal experiments. For transplantation, the model mice were divided into A-iSCs group, which received 2.5 × 10^5^ A-iSCs with a total volume of 2 ul (*n* = 8), the sham group, which received 2 ul of culture medium (*n* = 8), and the control group, which received 2.5 × 10^5^ astrocytes (*n* = 8). One week after MCAO induction, A-iSCs were transplanted into the contralateral side in the striatum region through the following stereotaxic coordinates: 1.0 mm anteroposterior, 3.0 mm mediolateral, and 5.0 mm dorsoventral from the bregma. A 26-gauge Hamilton syringe (Reno) was left in place for an additional 5 min to allow the stabilization of the transplanted cells.

### 2.12 Behavioral Testing

All animals were trained three times daily for three consecutive days under the same conditions before the tests to minimize variations among them. Before the footprint tests, the forelimbs and hindlimbs of the mice were dyed in red and blue color, which were allowed to walk across a narrow runway (80 cm in length and 4 cm in width) pre-marked on a piece of white paper (Yokota et al., 2017). The stride length, stride width, and paw rotation were, respectively, defined as the distance from the start to the end of a step with the back paws, the distance from the left to the right outermost toe as well as the angle between the axis of the back paws and the midline axis of the body. All the measurements were taken on each side through three consecutive steps for 8 weeks and averaged for comparison among groups. In the rotarod test, the mice were placed on a rotarod cylinder, in which the rod speed progressively increased from 4 to 40 r.p.m. for 2 min. The time for each mouse to remain on the cylinder was recorded weekly for consecutive 8 weeks, which was averaged for three trials (in seconds). A modified neurological severity score (mNSS) test was carried out immediately after injuries and neurological deficits were monitored in the following 8 weeks. The injuries were interpreted as follows: normal, 0 point; mild injuries, 1–9 points; moderate injuries, 10–19 points; severe injuries, 20–27 points; the most severe injuries, 28 points.

### 2.13 RT-PCR

The total RNA was extracted through the Trizol reagent and was used to synthesize cDNA according to the manufacturer’s instructions (Life Technologies). Subsequent PCRs were performed in a final volume of 20 µl containing 1 µl of cDNA and 1 µl of 10 pM primer in PCR Premix (Bioneer). Primer sequences and the reaction conditions used in this study are listed in Supplementary Table S1. For each sample, at least three independent samples were analyzed and the mRNA level of target genes was normalized to that of GAPDH for comparison.

### 2.14 Statistical Analysis

All the experimental results were presented as mean ± standard deviation (SD). Three independent experiments were carried out, and at least three samples were taken for statistical analyses in each test. The differences between the two samples were analyzed through a two-tailed unpaired *t*-test, while those among multiple groups of samples were analyzed through a one-way ANOVA test. A *p*-value < 0.05 was considered statistically significant.

## 3 Results

### 3.1 Generation of Induced Stem Cells From Mouse Astrocytes Through 3D Sphere Culture

**FIGURE 1:**
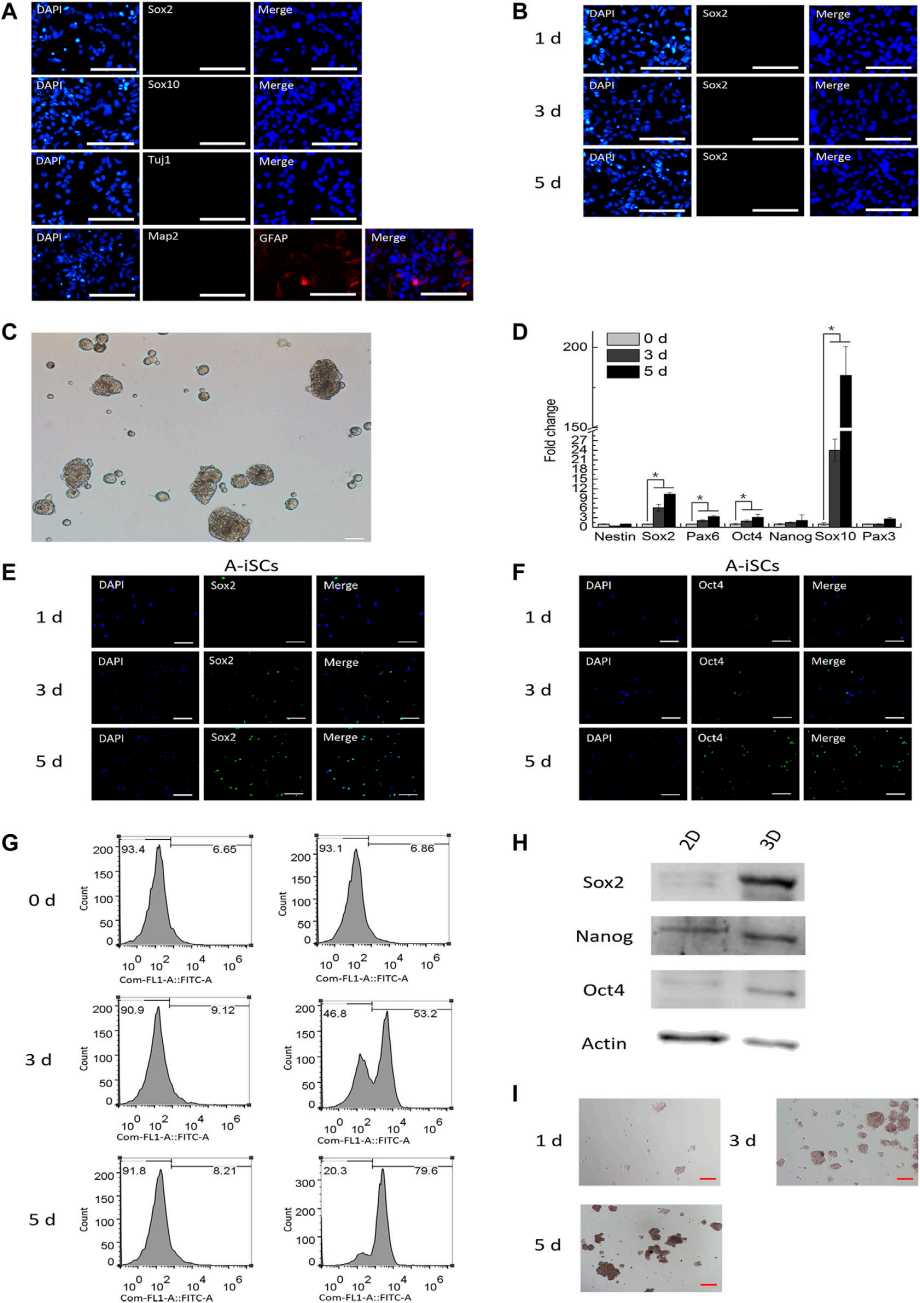
**(A)** Starting mouse astrocytes were immunopositive for GFAP but negative for Sox2, Sox10, Tuj1, and Map2. Scale bar: 50 μm. **(B)** Astrocytes in 2D culture were immuno-negative for Sox2. Scale bar: 50 μm. **(C)** Mouse astrocytes formed 3D spheres within 24 h on agarose surfaces. Scale bar: 50 μm. **(D)** RT-PCR analysis of stem cell-specific genes in mouse astrocytes in 3D spheres over time. **(E)** Expression of Sox2 in mouse astrocytes in 3D spheres over time. Scale bar: 50 μm. **(F** Expression of Oct4 in mouse astrocytes in 3D spheres over time. Scale bar: 50 μm. **(G)** Expression of Sox2 in mouse astrocytes in 3D spheres (flow cytometry). **(H)** Western blotting of stem-specific markers in 2D- and 3D-cultured astrocytes. **(I)** Alkaline phosphatase staining for mouse astrocytes in 3D spheres over time. Scale bar: 50 μm.

The culture of mouse astrocytes was not contaminated by neural stem cells or neural crest progenitors as shown by negative staining of monolayered ASTs for Sox2 and Sox10 (Figure 1A). Additionally, the mouse astrocytes in the culture were immune-positive for astrocyte marker GFAP but were immune-negative for neuron markers Tuj1 and Map2 (Figure 1A). These observations confirmed that the astrocytes were pure, without any contamination of stem cell-like cells during isolation or cultivation. The expression of Sox2 was not detected when ASTs were cultured on 2D monolayer dishes in an AST expansion medium over a period of 5 days (Figure 1B). The above data illustrated that in 2D environments, ASTs did not present stem cell-associated features. When mouse ASTs were cultured on low-attachment dishes prepared by coating with 0.5% of agarose in an AST expansion medium, they tended to form 3D spheres within 24 h (Figure 1C), and the structures could be maintained for up to a couple of weeks. Compared with monolayer ASTs, ASTs in 3D spheres showed an upregulated expression of a series of stem cell-associated genes, such as Sox2, Pax6, Oct4, Nanog, Sox10, and Pax3 (Figure 1D). The expression of stem cell-associated genes reached the highest level on day 5 in 3D spheres, which declined thereafter (data not shown). Immunostaining of ASTs in 3D spheres confirmed that they were positive for Sox2 (1, 40 and 86% on Day 1, Day 3, and Day 5, respectively) (Figure 1E) and Oct4 (1, 30 and 70% on Day 1, Day 3, and Day 5, respectively) (Figure 1F) when cultured on low-attachment dishes, in contrast to ASTs in 2D culture (Figure 1A). These results indicate that the expression level of stem cell-associated genes increases over time in 3D cultivation. Similarly, it was revealed through a FACS analysis that the expression of Sox2 was not detectable in the 2D culture (0-day incubation) but 54% (3-day incubation) and 66% (5-day incubation) of ASTs in 3D spheres were found positive for Sox2 (Figure 1G). It was shown through Western blotting analysis that the level of Sox2, Oct4, and Nanog of ASTs was significantly enhanced within 3D environments compared to the 2D culture (Figure 1H). Additionally, it was found that the ASTs in 3D spheres started to become alkaline phosphatase (ALP)-positive after 3 days of incubation and the positivity even increased on Day 5 (Figure 1I). The immunostaining, flow cytometry, Western blotting, and ALP assay of A-iSCs collectively confirmed that the expression level of stem cell-associated genes was highly consistent with the RNA expression profiles. Taken together, it was confirmed that ASTs gradually acquired the stem cell features and were channeled into stem cell-like cells (denoted as A-iSCs) after aggregation culture in 0.5% agarose.

### 3.2 3D Microenvironment-Induced Dramatic Changes of Gene Expression Profile in ASTs

**FIGURE 2:**
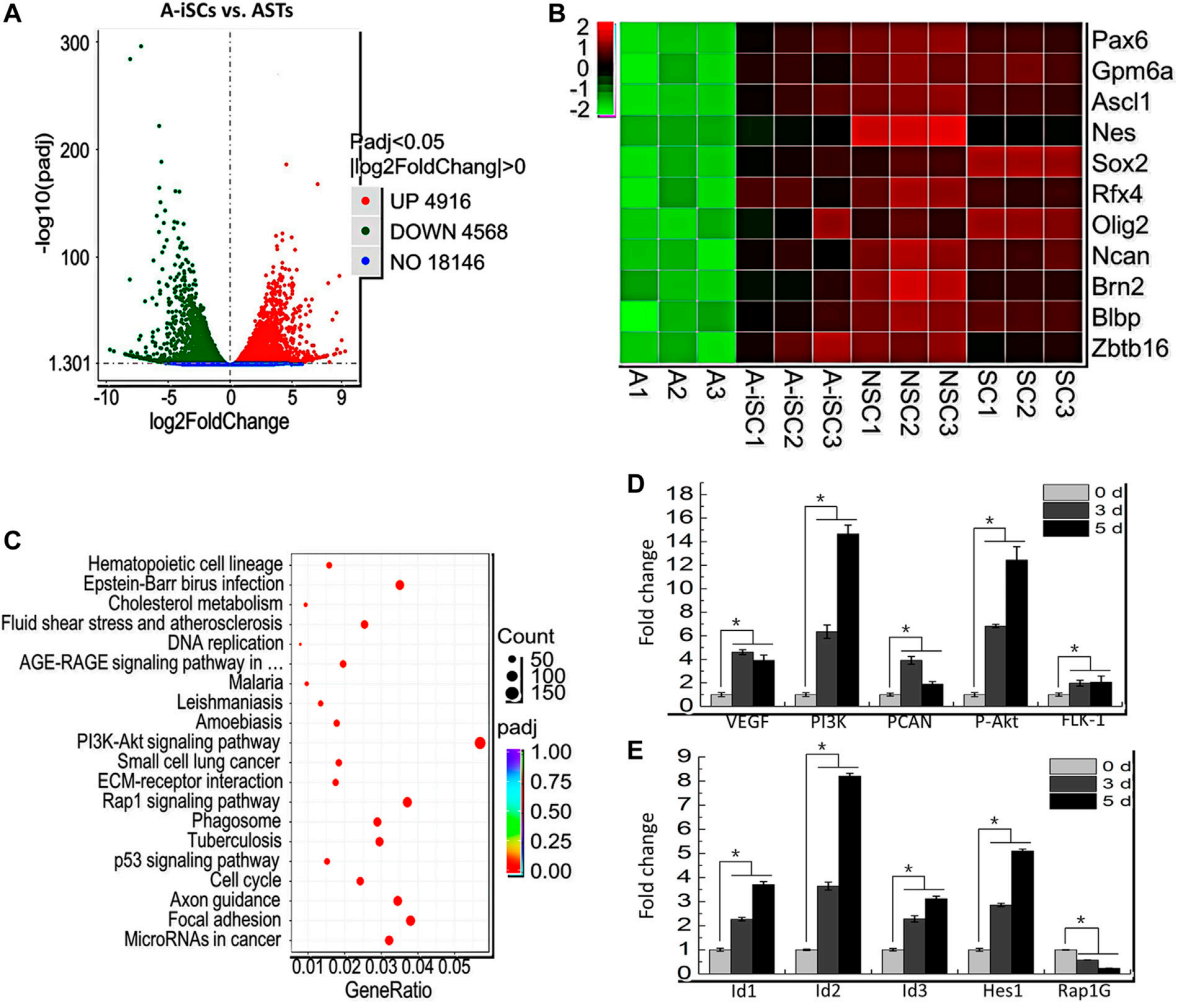
**(A)** Volcano plot of differentiated genes between ASTs and A-iSCs. **(B)** Heat map of representative high-expression genes in A-iSCs compared to ASTs, P56-NPCs, and P0-NPCs. **(C)** Pathways involved in A-iSCs compared with ASTs in KEGG analysis. **(D)** RT-PCR analysis of VEGF, PI3K, PCNA, P-Akt, and FLK-1 in A-iSCs at specific time intervals. *p < 0.05. **(E)** RT-PCR analysis of Id1, Id2, Id3, Hes1, and Rap1G in A-iSCs at specific time intervals. *p < 0.05.

To elucidate the mechanism involved in the induction of ASTs in 3D cell spheres, global gene expression profiles of ASTs (0-day incubation, negative control) and A-iSCs (5-day incubation) were characterized by RNA-sequencing, with the profiles of brain-derived P56-NPCs and P0-NPCs as positive controls. After trimming for quality, 93.48, 104.42, 111.56, and 116.02 million of RNA-seq clean reads were obtained for ASTs, A-iSCs, P56-NPCs, and P0-NPCs, respectively, and the quality of sequencing data was summarized in Supplementary Table S2. As shown in the heatmap (Supplementary Figures S1A,B), marked changes in the global gene expression pattern were observed between ASTs and A-iSCs. The volcano plot of the differentially expressed genes (DEGs) between A-iSCs and ASTs showed remarkable differentiations with 4916 upregulated genes and 4568 down-regulated genes (*p* < 0.05) in A-iSCs in comparison to those in ASTs (Figure 2A). The hierarchical cluster analysis of stem cell-associated genes, such as Sox2, Pax6 and Oilg2, confirmed that the beginning ASTs were truly not contaminated by the stem cells, but the stem cell-associated genes were remarkably upregulated in 3D spheres (Figure 2B). The expression patterns of A-iSCs associated with NPCs were closely similar to those of mouse P56-NPCs or P0-NPCs (Figure 2B).

To gain a further sight into the identity of A-iSCs, functional and pathway enrichment analyses, including GO and KEGG, were performed through cluster Profiler R package. The most enriched processes in A-iSCs included the regulation of peptide secretion, protein secretion and cell morphogenesis (BP category), cell-substrate adhesion, glutamatergic synapse (CC category), cell adhesion molecule binding, integrin binding, and actin binding (MF category) compared with ASTs (Supplementary Figure S1C). It was revealed through a KEGG analysis that the all DEGs in A-iSCs vs ASTs were mainly enriched in several signaling pathways, including PI3K-Akt signaling pathway, Rap1 signaling pathway, focal adhesion, microRNA in cancer, and p53 signaling pathway (Figure 2C). Among the GO categories and KEGG pathways, PI3K-Akt signaling pathway (Shioda et al., 2009) and Rap1 signaling pathway (Niola et al., 2012) were exceptionally crucial as embryonic morphogenesis and adult hair regeneration were regulated through them. In order to confirm the involvement of these two pathways in the conversion of ASTs into A-iSCs, RT-PCR analysis of five critical genes associated with PI3K-Akt signaling pathway, including VEGF, PI3K, PCNA, P-Akt, and FLK-1, and another four critical genes associated with Rap1 signaling pathway, including Id1, Id2, Id3, and Rap1Gap, as well as one critical genes associated with the Notch signaling pathway, including Hes1, were carried out. It was found that the expression patterns (Figures 2D,E) were consistent with RNA-seq results, and all the genes were upregulated in 3D cell spheres over time, except Rap1GAP. Based on the abovementioned observation, we assume that the A-iSCs conversion in this scenario is composed of heterogeneous signaling pathways including PI3K-Akt signaling pathway, Rap1 signaling pathway, and Notch signaling pathway.

### 3.3 *In Vitro* Differentiation of A-iSCs

**FIGURE 3:**
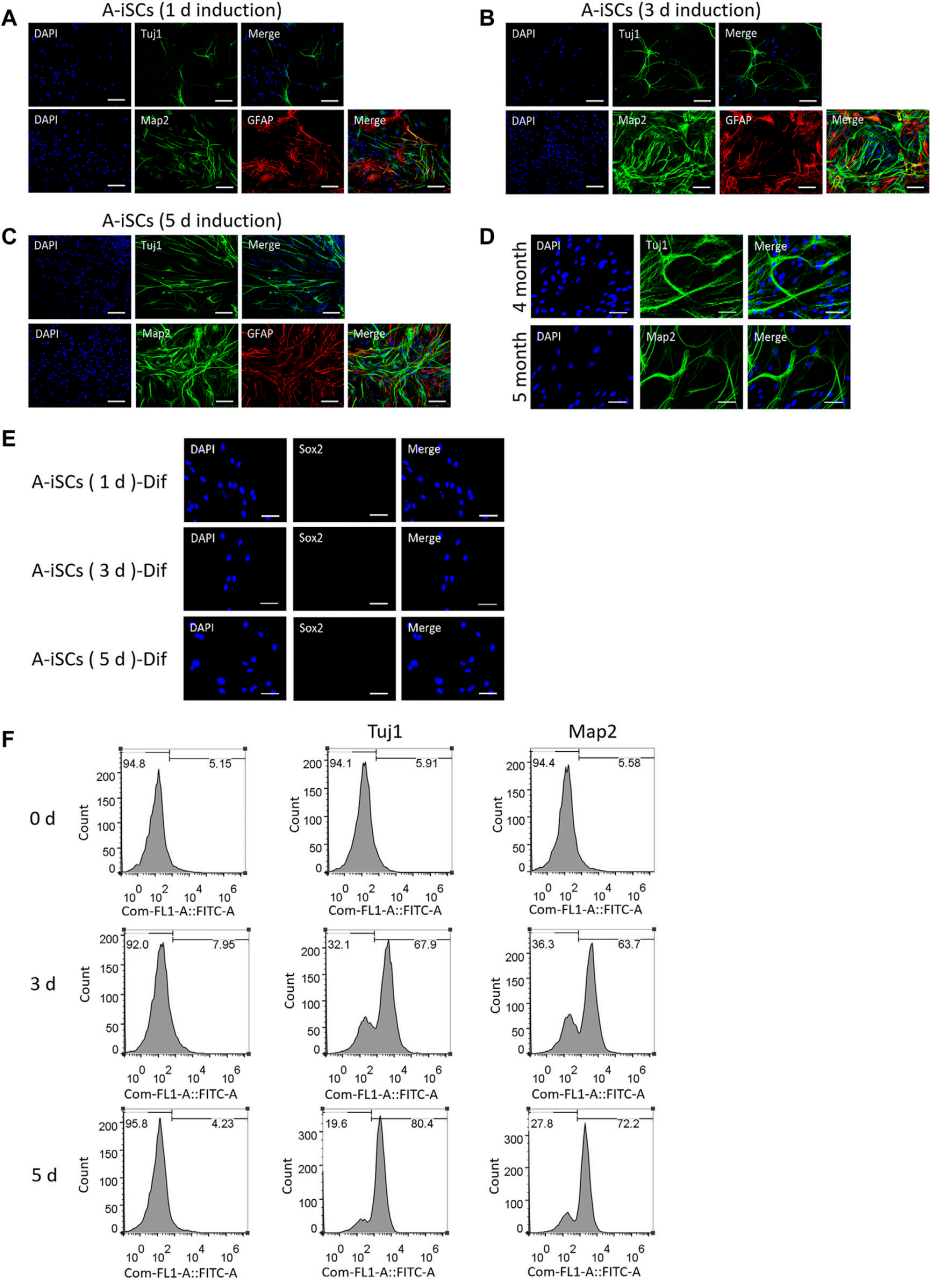
*In vitro* differentiation of A-iSCs derived from ASTs induced in spheres for 1 day **(A)**, 3 days **(B)** and 5 days **(C)**, respectively. Scale bars of **(A–C)**: 100 μm. **(D)** Neurons converted from A-iSCs survived for >5 months. Scale bar: 50 μm. **(E)** Expression of Sox2 in differentiated A-iSCs derived from ASTs induced in spheres for 1, 3, and 5 days, respectively. A-iSCs (1 day)-Dif: Differentiation of A-iSCs derived from ASTs induced in spheres for 1 day; A-iSCs (3 days)-Dif: Differentiation of A-iSCs derived from ASTs induced in spheres for 3 days; A-iSCs (5 days)-Dif: Differentiation of A-iSCs derived from ASTs induced in spheres for 5 days. Scale bar: 50 μm. **(F)** Expression of neuron-specific markers in differentiated A-iSCs derived from ASTs induced in spheres for 0 (2D culture), 3 and 5 days, respectively.

As displayed in Figures 1D–I, A-iSCs at various induction stages (including induction for 1 day, 3, and 5 days, respectively) express representative stem cell markers, meanwhile, they are not positive for markers of neurons. When trypsinized and seeded onto poly-ornithine/laminin (POL)-coated plates and cultured in differentiation media, they started to exhibit neural lineage cells-like morphologies, with long and branching processes (Figures 3A–C). The differentiation potential of A-iSCs was positively correlated to the incubation time period of ASTs in 3D spheres. Relatively, the A-iSCs incubated for 5 days were easier to be converted to Tuj1^+^ or Map2^+^ cells compared with those incubated for just 1 day. Specifically, 25.76, 56.73 and 75.33% of the A-iSCs (1 day)-, A-iSCs (3 days)-, and A-iSCs (5 days)-derived cells were stained, which were respectively positive for Tuj1 after differentiation. 21.52, 53.19, and 68.67% of the A-iSCs (1 day)-, A-iSCs (3 days)-, and A-iSCs (5 days)-derived cells were stained, which were respectively positive for Map2 after differentiation. The neurons converted from the A-iSCs survived longer than 5 months in the culture and robust axons and dendrites were developed by the end of 5 months (Figure 3D). Regardless of the differentiation potential of A-iSCs, they were negatively stained with Sox2 after differentiation induction (Figure 1E). In addition to the immunostaining evidences, FACS analysis of Tuj1 and Map2 revealed a negative expression of them in the post-differentiation-induction monolayer ASTs, but 71.28 and 85.59% of the A-iSCs (3 days)- and A-iSCs (5 days)-derived cells were Tuj1^+^ after differentiation induction, respectively (Figure 1F). Additionally, 48.38 and 71.19% of the A-iSCs (3 days)- and A-iSCs (5 days)-derived cells were Map2^+^ after differentiation induction. These results of immunostain and FACS analysis indicate that the neuronal phenotypes of A-iSCs can be achieved and the differentiation potential of A-iSCs is highly correlated to the stemness of the cells with the A-iSCs (5 days) possessing the highest degree of stemness and differentiation potential to neurons.

**FIGURE 4:**
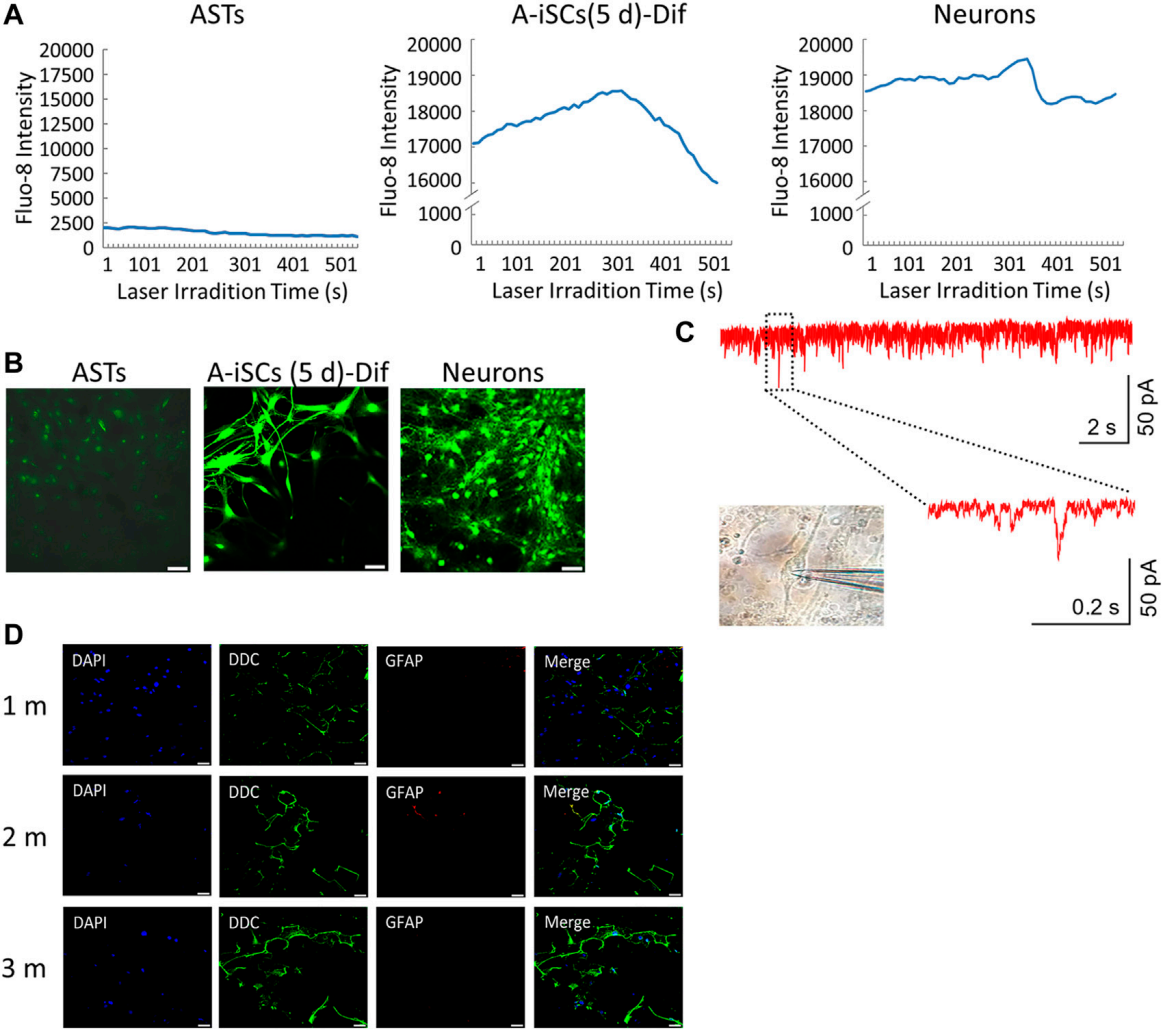
Functional analyses of A-iSCs-converted neurons. **(A)** Comparison of the patterns of synchronized Ca^2+^ spikes among monolayer ASTs, A-iSCs-derived neurons, and naive neurons. **(B)** Fluorescent calcium imaging of monolayer ASTs, A-iSCs-derived neurons, and naive neurons. Scale bar: 50 μm. **(C)** Representative traces showing spontaneous synaptic events in A-iSCs-derived neurons. **(D)** A-iSCs-derived neurons showing an increased number of synaptic puncta along the dendrites over time. m, month. Scale bar: 50 μm.

Fura-2 calcium imaging and recording of synchronized Ca^2+^ spikes were employed to verify the functions of the A-iSC-derived neurons. The patterns of synchronized Ca^2+^ spikes, such as peak signals and durations, were quite similar between A-iSC-derived neurons and naive neurons, both of which were in contrast to the pattern of monolayered ASTs (Figure 4A). The fluorescent imagining results confirmed the enhancement of calcium signals in A-iSC-derived neurons and the cells were functionally networked (Figure 4B) similar to naive neurons. Patch clamp technique was then employed to examine the electro-physiological features of A-iSC-derived neurons. In current clamp model, some neurons were able to fire an action potential (Figure 4C), demonstrating their membrane excitability. In addition, some neurons expressed dopaminergic neuron marker DDC after cultivation for 1 month, 2 months, or 3 months (Figure 4D). Therefore, A-iSCs (5 days) can be efficiently differentiated into functional neurons, which possess a high potential of generating dopaminergic neurons.

### 3.4 *In Vivo* Neuronal Differentiation of Transplanted A-iSCs

**FIGURE 5:**
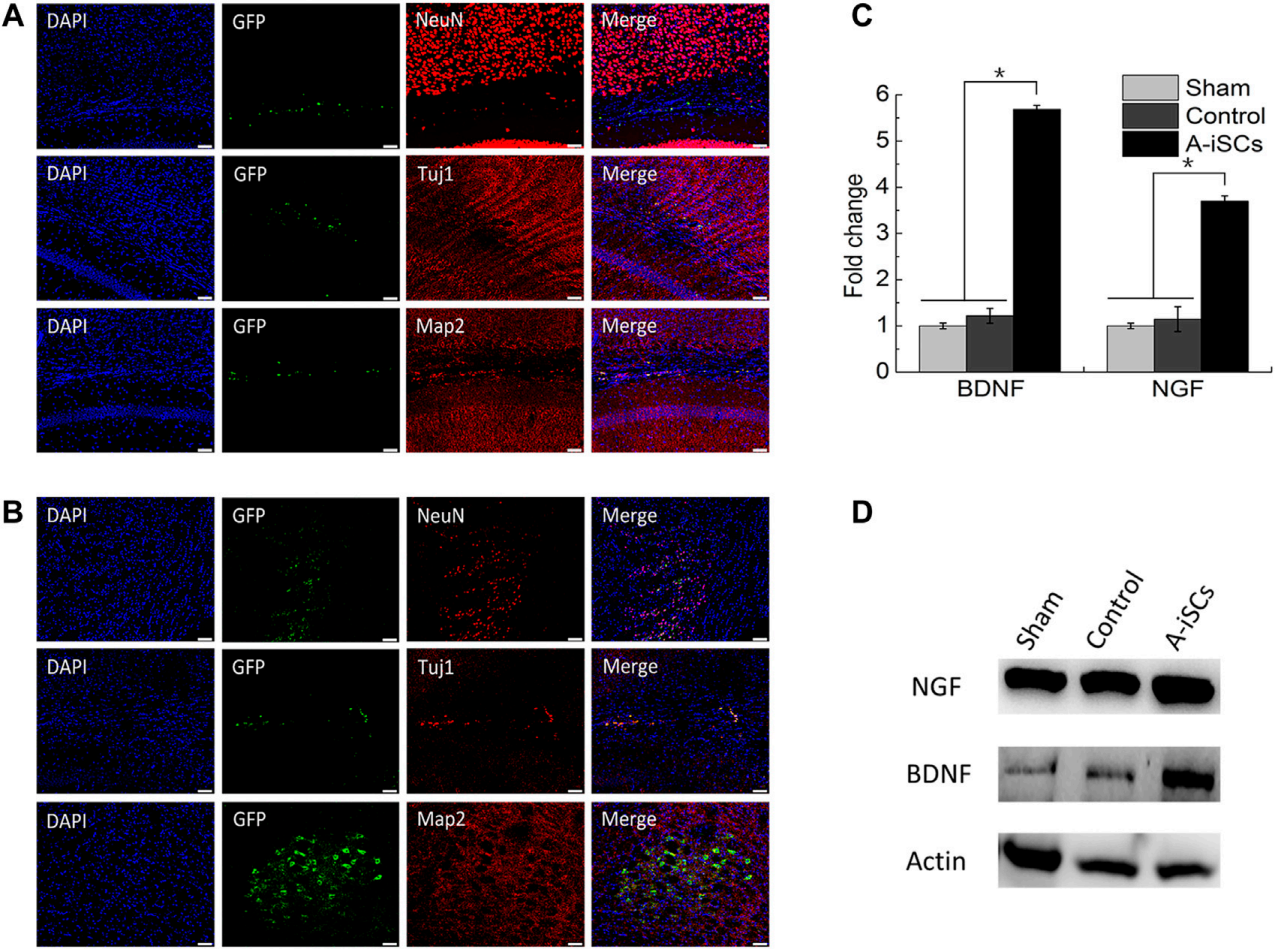
Immunostaining of NeuN, Tuj1, and Map2 in A-iSCs transplanted into the brains of normal mice **(A)** and MCAO mice **(B)**. Scale bars of **(A,B)**: 50 μm. **(C)** RT-PCR for BDNF and NGF of the MCAO mice through A-iSCs transplantation in comparison to sham and control groups at 8 weeks. *p < 0.05. **(D)** Western blotting for BDNF and NGF of the MCAO mice through A-iSCs transplantation in comparison to sham and control at 8 weeks.

MCAO model was employed to evaluate the therapeutic potential of A-iSCs (Supplementary Figure S2). A-iSCs pre-labeled with GFP (Supplementary Figure S3) were transplanted into the brain of mice, and the survival, as well as differentiation capability of the cells, was analyzed after 4 weeks. In the brain of normal mice, a small number of GFP-positive cells were detected and positive for neural precursor/stem cell markers (NeuN, Tuj1, and Map2) (Figure 5A), demonstrating the *in vivo* differentiation potential of A-iSCs in the brains of normal mice. In the brain of MCAO mice, a larger percentage of the cells were found positive for NeuN, Tuj1, and Map2 (Figure 5B) compared with the cases of normal mice, demonstrating the *in vivo* differentiation capability of A-iSCs in the circumstance of MCAO. As the brain-derived growth factor (BDNF) and nerve growth factor (NGF) play vital roles in repairing neural deficiencies, we tested the expression of BDNF and NGF by RT-PCR and WB at 8 weeks post-transplantation of A-iSCs. RT-PCR showed that the expression of BDNF and NGF was significantly increased in the A-iSCs group compared to sham and control groups (*p* < 0.05) (Figure 5C). The results of the Western blotting assay were well correlated with the RT-PCR results. The A-iSCs group showed higher expression levels of BDNF and NGF than the sham group and control groups (Figure 5D). These results indicate that the transplantation of A-iSCs significantly increases the expression of BDNF and NGF, and the enhancement may consequently promote nerve regeneration in MCAO model mice.

### 3.5 A-iSCs Transplantation Improved the Functional Recovery of MCAO Mice

**FIGURE 6:**
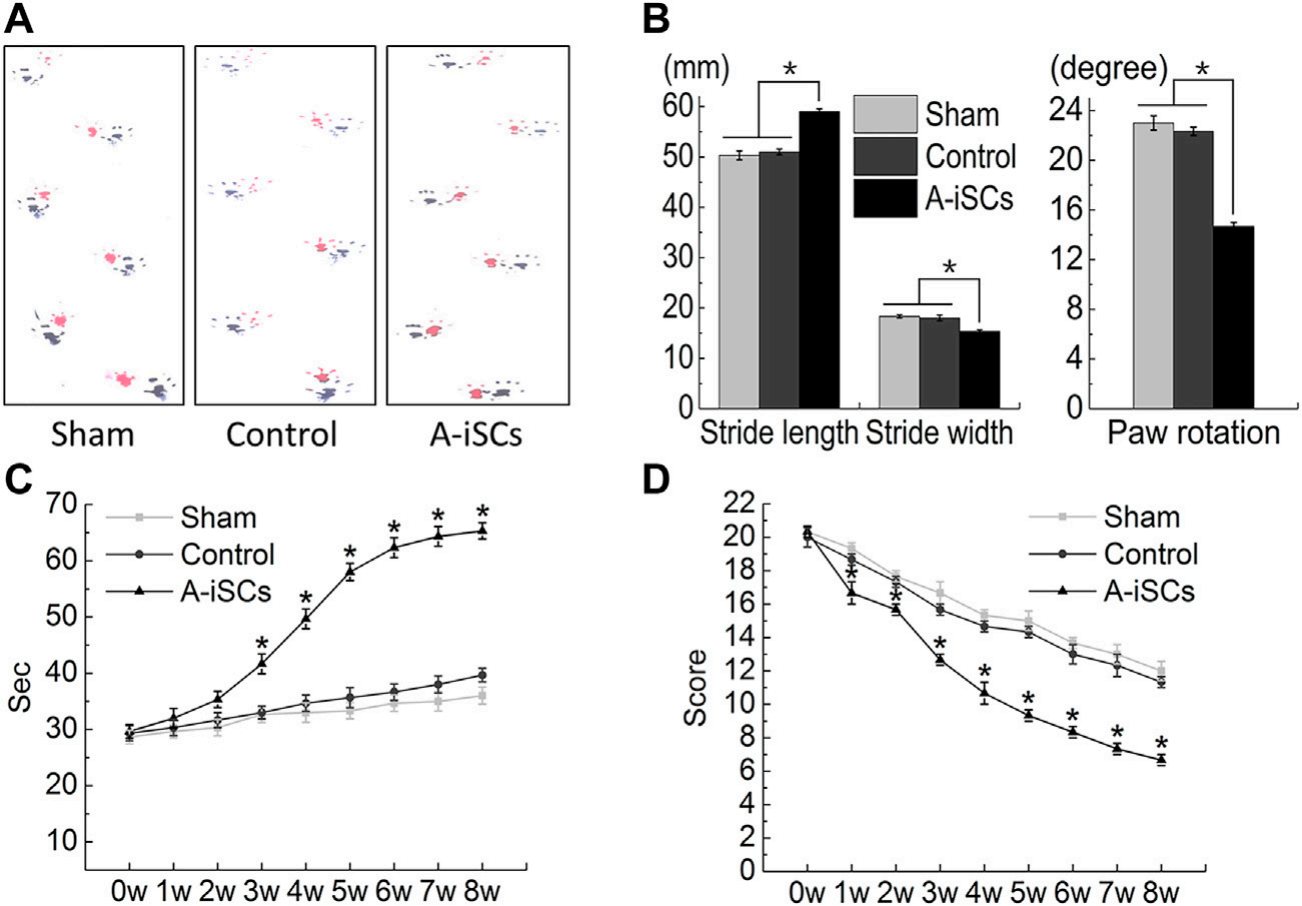
Behavioral improvement following the transplantation of A-iSCs into MCAO mice **(A–D)**. **(A,B)** The results of the footprint analyses and grip walk test of the mice with A-iSCs transplantation in comparison to those in sham and control groups at 8 weeks. **(C)** Rotarod testing of the mice with A-iSCs transplantation in comparison to those in sham and control groups over 8 weeks. **(D)** Modified neurological severity score (mNSS) testing of the mice with A-iSCs transplantation in comparison to those in sham and control groups over 8 weeks. The data are presented as mean ± SD. *p < 0.05.

The functional recovery of MCAO mice after A-iSCs transplantation was assessed by the footprint analysis, rotarod testing, and open-field testing based on the Basso Mouse Scale (BMS) score (Figure 6). In terms of footprints, longer and narrower strides, as well as smaller rotation angles, were noticed in the A-iSCs group than in the sham and control groups (*p* < 0.05) at 8 weeks in MCAO mice (Figures 6A,B). The rotarod testing results showed a slight increase in the A-iSCs groups compared with the sham and control groups (*p* < 0.05) at 2 weeks in MCAO mice (Figure 6C), and this improvement gradually became more prominent since Week 3 till the end of testing (8 weeks). As for the modified neurological severity scores, we observed an obvious improvement in the A-iSCs group at 1-week post-transplantation compared with the sham and control groups (*p* < 0.05), and the extent of this improvement became even more significant after 3 weeks (*p* < 0.05) (Figure 6D). As the functional performances were consistent in almost all physiological examinations (Figure 5), it was reasonable to ascribe the functional recovery in MCAO mice to the transplantation of A-iSCs.

## 4 Discussion

Diseases or injuries of adult mammalian neural networks tend to result in neurological deficiencies as hosts in the central nervous system limit NPCs, which remarkably bottlenecks its regenerative progress. Therefore, securing a readily available source of transplantable neural cells with an identical genetic background to patients is of great significance for cell-based neurological disease therapies. NPCs can self-renew and differentiate into neurons, astrocytes as well as oligodendrocytes, which are thus well-recognized as promising somatic stem cells for neural regeneration. However, it is not practical to obtain a sufficient number of NPCs without sacrificing tissues in the subgranular zone of the hippocampus or the ventricular subependymal zone. A series of studies have demonstrated the possibility of direct reprogramming of fibroblasts into functional neurons driven by neural tissue-specific factors (Caiazzo et al., 2011; Pfisterer et al., 2011; Son et al., 2011; Liu et al., 2012), but the involvement of ectopic transgenes or chemicals limits their practical therapeutic applications due to safety concerns. As ASTs are quite plentiful in the brain and are particularly over-proliferated in some pathological scenarios, the successful induction of ASTs into NPC-like cells would potentially provide a sufficient number of NPCs for the replenishment of neurons or other neural cell types.

Here we show that through 3D sphere culture on agarose-coated dishes, mouse ASTs were induced into NPC-like cells (A-iSCs) without the involvement of any exogenous DNA, RNA, or proteins. In this study, ASTs were isolated from the brain tissues based on recognized protocols. It was confirmed by immunostaining assay of ASTs that the starting ASTs were not contaminated by NPCs or neurons. Based on RT-PCR, immunostaining, FACS, and Western blotting results, we found that the upregulation of stem cell-associated genes was significant when the ASTs were cultured in cell spheres (induced by agarose surfaces) for 3 or 5 days. It is well accepted that the microenvironment of stem cells plays a big role in regulating the fate of the resident cells (Zhang et al., 2018), which can direct the cells to remain in a quiescent state, undergo self-renewal, exit the niche (Vazin and Schaffer, 2010), or mediate the dedifferentiation reprogramming of ectopic, or differentiated cells to corresponding stem cell phenotypes (Hendrix et al., 2007). We speculate that a microenvironment prone to the dedifferentiation of ASTs happens to be established through 3D sphere culture. RNA-sequencing results confirmed the acquisition of key stem cell features in 3D cell spheres. Based on global gene expression profiles of A-iSCs vs ASTs, we choose the PI3K-Akt signaling pathway (Shioda, et al., 2009; Landgren and Curtis, 2011), Rap1 signaling pathway (Niola, et al., 2012), and Notch signaling pathway (Bai et al., 2007) to analyze AST conversion mechanism as these three pathways are exceptionally crucial for the neural development. Our results suggest that a series of critical genes were activated along the PI3K-Akt pathway through the 3D spherical culture of ASTs on 0.5% agrarose such as VEGF, PI3K, P-Akt, and FLK-1. Therefore, we believe that PI3K-AKT may be one of the pathways facilitating the conversion of ASTs to A-iSCs. Along the Rap1 signaling pathway, a series of critical genes were activated in the 3D microenvironment, such as Id1, Id2, and Id3, which act redundantly to drive stemness (Jankovic et al., 2007). RAP1 is a key mediator of cell adhesion, which positively regulates integrin signaling (Boettner and Van Aelst, 2009). The broad activation of Id proteins and the inhibition of RAP1 may be a mechanism used by ASTs to acquire stem cell-associated features. Along the Notch signaling pathway, Hes1, one of the genes highly expressed in NPCs, was activated in A-iSCs (Bai et al., 2007), sharing a similar expression pattern as Ids. All the above results imply that the neural precursor cells induced by 3D microenvironment culture were accompanied by the increase of PI3K-Akt, Rap1, and Notch signaling. On the other hand, through KEGG analysis of differential genes in neural precursor cells and mouse astrocytes in RNA-seq, a significant increase was also shown in PI3K-Akt, Rap1, and Notch signaling during this process. The rise of PI3K-Akt, Rap1, and Notch-related genes has been confirmed to play an important role in driving the expression of stemness-related genes. Taken together, our results strongly support the notion that A-iSCs were possibly derived through multiple mechanisms, including PI3K-Akt signaling pathway, Rap1 signaling pathway, and Notch signaling pathway.

In the animal model of MCAO, the transplantation of NPCs was proven to be effective for ameliorating behavioral deficits (Mack, 2011). Recently, there has been more involvement of stem cells, such as NPCs (Pandamooz et al., 2018), ESC-derived cells (Iwai et al., 2015), and MSCs (Ye et al., 2018) so as to mitigate inflammation and replenish damaged neural cells in animal models of SCI (Tetzlaff et al., 2011; Mothe and Tator, 2013; Volarevic et al., 2013) and PNI (Goel et al., 2009; Cotter et al., 2010; Tang et al., 2013). Despite these advancements in cell therapy strategies, the functions of particular cells of interest in one of the animal models are investigated in most of the studies on cell-therapies, e.g., in most of the cases, PNI overlooks the complexity of the mammalian neural system and excludes the possibility of general application of particular cells in the whole neural system. Herein the current study, we have demonstrated that the local transplantation of A-iSCs successfully ameliorated all kinds of the lesions in MCAO model. At the sites of lesions, a portion of loaded A-iSCs (GFP-labeled) was found positive for NeuN, Tuj1, and Map2 in the A-iSCs treated group among the three injured models (experimental data not shown for models of spinal cord injury and peripheral nerve injury) indicating the development of A-iSCs toward neuronal phenotypes. More remarkably, a significant functional improvement was detected following the receipt of A-iSCs. The gradual sensorimotor improvement after over 8 weeks with A-iSCs suggested the true recovery-enhancing effect of A-iSCs in all three models (experimental data were not shown for models of spinal cord injury and peripheral nerve injury). As a portion of the transplanted A-iSCs expressed a series of mature neuronal markers, such as NeuN and Map2, at the injured sites, it was likely that the functional improvement was ascribed to the derivation of neurons from some of the A-iSCs, according to a hypothesis proposed in a previous study in which grafted mouse ESC-NPCs differentiated into mature neurons 12 weeks after transplantation. In addition, neural cell replacement mechanisms, several other possible explanations for the efficacy of engrafted cells have been suggested, such as remyelination, growth support, neuroprotection, and immunomodulation (Volarevic et al., 2013). It was found in our study that the expression of BDNF and NGF at gene and protein levels in the lesion tissues of A-iSCs group significantly increased compared to those of sham and control groups (*p* < 0.05) in all the injured models (Figure 5). In this sense, the transplanted A-iSCs possibly function in an indirect manner in regenerating nerves, besides being directly converted into new neurons. The investigation into exact mechanisms directing ASTs into A-iSCs followed by the regeneration of neurons are warranted in the future.

Unlike the tissue engineering-oriented hydrogels we previously reported for cell loading (Zhong et al., 2015; Li et al., 2017; Zhang, et al., 2018; Xie et al., 2020), the agarose surfaces in the current study were solely employed to induce the conversion of phenotypes of cells by forming cell spheres. The material itself is not entrapped into cell spheres or transplanted into animal bodies, so the agarose surfaces would minimize safety concerns, which would facilitate the translational medicine research projecting clinical applications of A-iSCs in the near future.

## 5 Conclusion

The proof-of-concept study demonstrates that mouse astrocytes can be induced into NPC-like cells through physical induction by culturing on agarose substrates without using any exogenous genes, RNAs, or proteins. The astrocytes agglomerated into 3D spheres exhibited similar morphological and molecular features to those of NPCs, the characteristics of self-renewal, and differentiation into functional neurons. The derived neurons still survived in a 2D culture environment after 5 months of monitoring. 3D sphere culture of astrocytes provides another perspective for understanding the regulation of cell stemness and sheds light on developing a safe as well as practical technique to obtain NPC-like cells through physical approaches. Animal experiments indicated that the local transplantation of A-iSCs promoted nerve regeneration and improved nerve functions in the mouse MCAO model. Therefore, our findings develop a cell platform to understand the trans-differentiation of cells among neural lineages and to evaluate the therapeutic potentials of the NPC-like cells derived in models with neural injuries.

## Data Availability

The datasets presented in this study can be found in online repositories. The names of the repository/repositories and accession number(s) can be found below: GEO database, accession numbers GSE197711, and GSM5928599–GSM5928610.
