# Supplementary figures and images for: Derivation of Stem Cell-like Cells From Spherical Culture of Astrocytes for Enhanced Neural Repair After Middle Cerebral Artery Occlusion
Source: Front Bioeng Biotechnol. 2022 Apr 4;10:875514. doi: 10.3389/fbioe.2022.875514 (PMC9013960; doi:10.3389/fbioe.2022.875514)

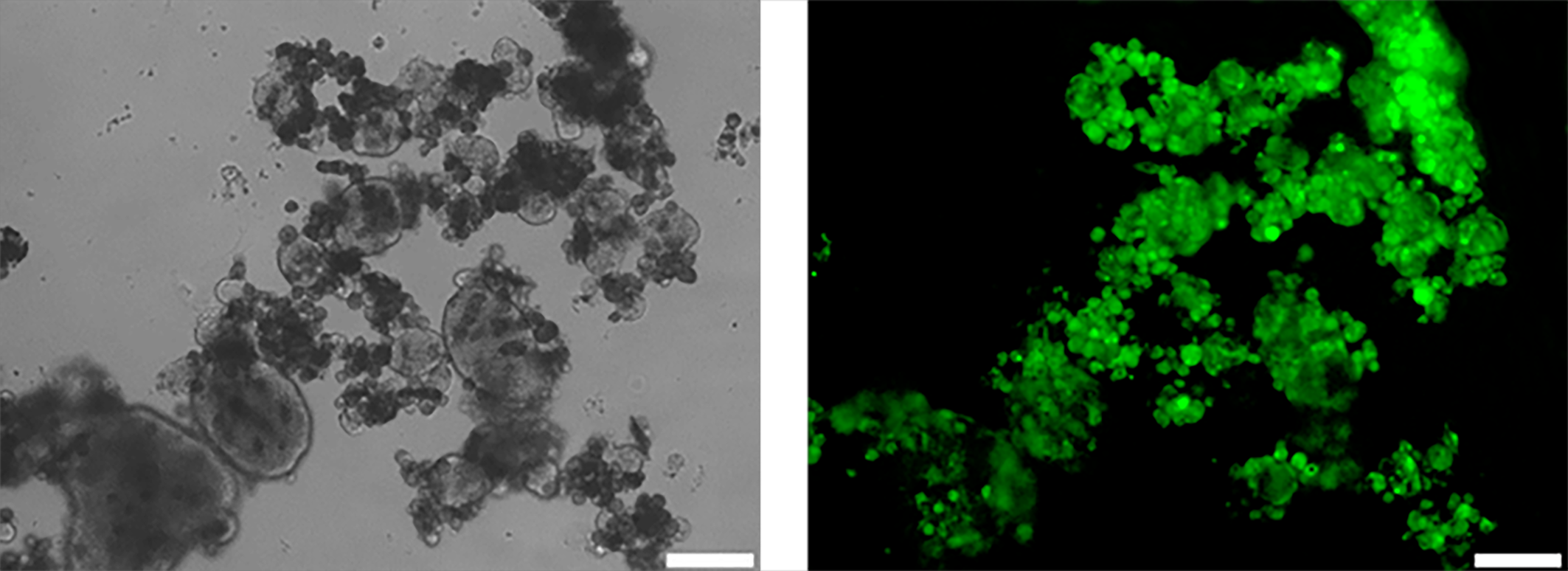

Supplement: Supplementary file 1 [file Image3.TIF]

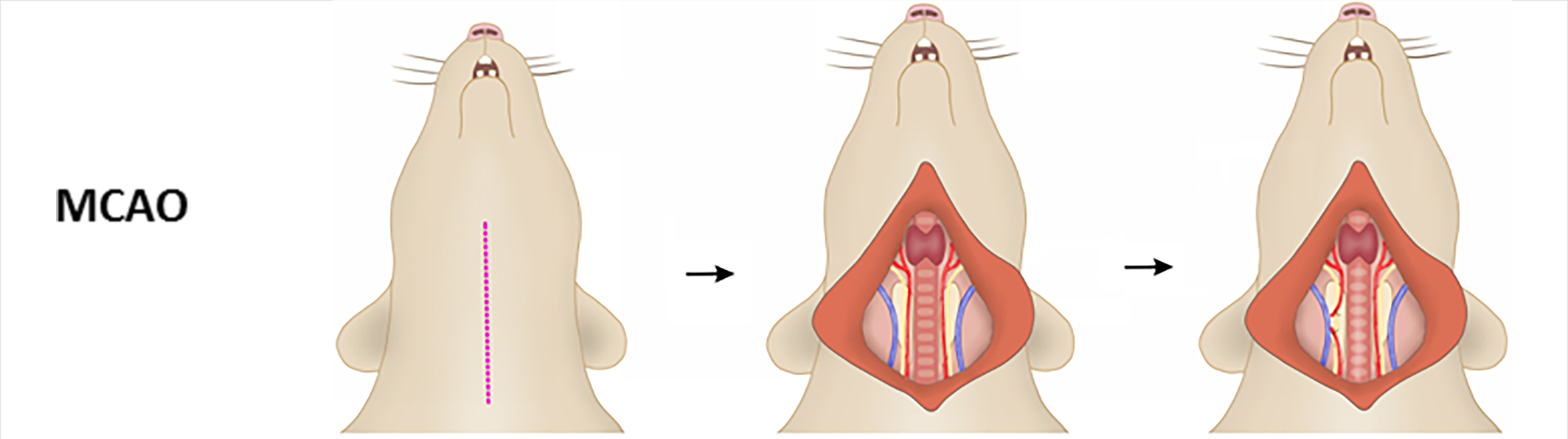

Supplement: Supplementary file 2 [file Image2.TIF]

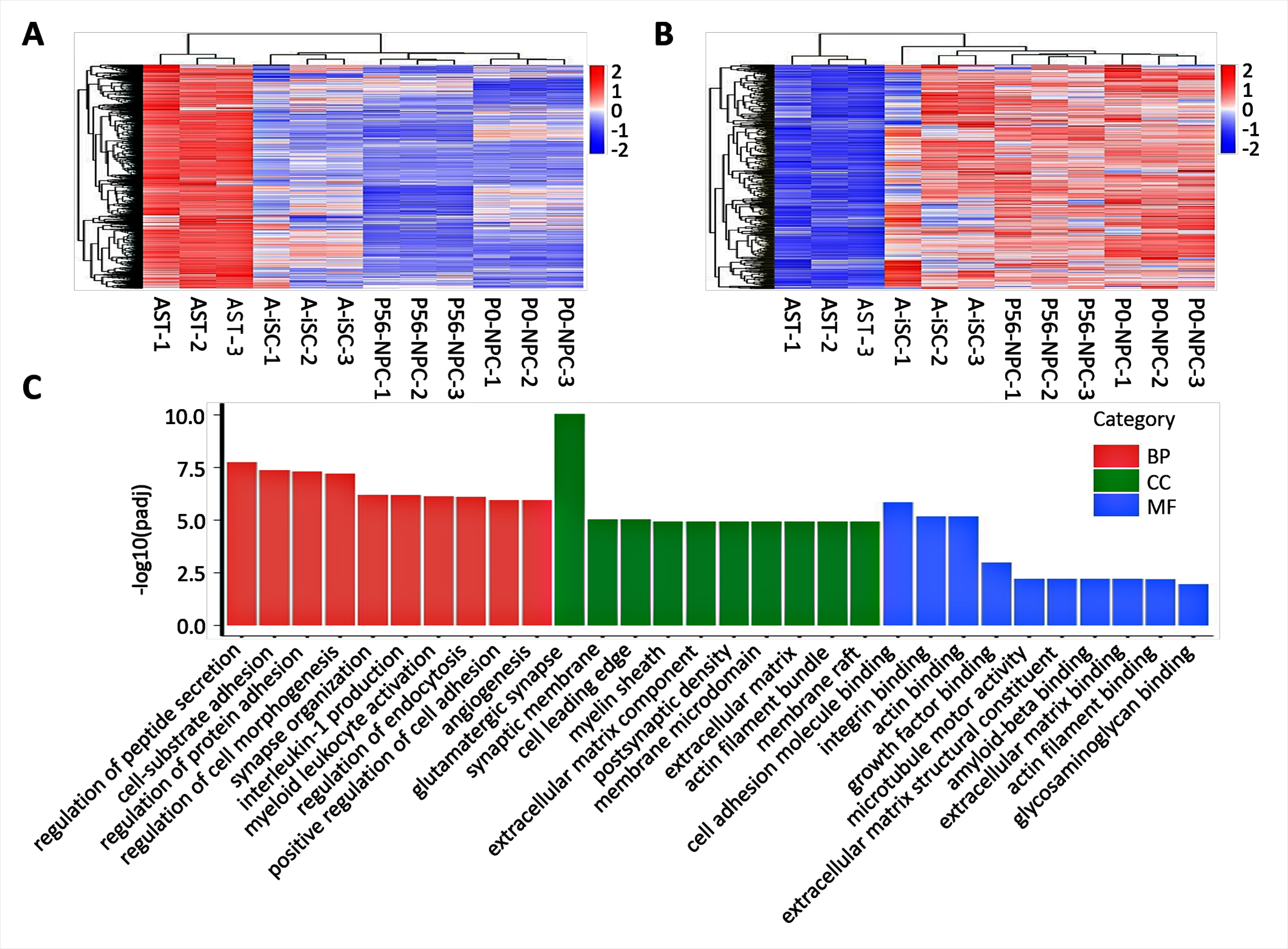

Supplement: Supplementary file 3 [file Image1.TIF]
