# Supplementary material for: Derivation of Stem Cell-like Cells From Spherical Culture of Astrocytes for Enhanced Neural Repair After Middle Cerebral Artery Occlusion
Source: Front Bioeng Biotechnol. 2022 Apr 4;10:875514. doi: 10.3389/fbioe.2022.875514 (PMC9013960; doi:10.3389/fbioe.2022.875514)
Supplement: Supplementary file 4 [file DataSheet1.docx]

**Additional information:**

**Table S1. Primer sequences for quantitative real-time PCR used in this study.**

| **Gene name** | **Forward primer (5’-3’)** | **Reverse primer (5’-3’)** |
| --- | --- | --- |
| Nestin | GGCATCCCTGAATTACCCAA | AGCTCATGGGCATCTGTCAA |
| Sox2 | AGCAACGGCAGCTACAGCATGA | CTGCGAGCTGGTCATGGAGTTGTA |
| Pax6 | TGCCCTTCCATCTTTGCTTG | AGCCTCAATCTGCTCTTGGGT |
| 4-Oct | CGACCGCCCCAATGC | TGGGACTCCTCGGGAGTTG |
| Nanog | AGGCCTGGACCGCTCAGT | AGTTATGGAGCGGAGCAGCAT |
| Sox10 | GGAGGTTGCTGAACGAAAGTG | GAGCCTCTCAGCCTCCTCAA |
| Pax3 | ATGGTTGCGTCTCTAAGATCCTG | GCGTCCTTGAGCAATTTGTC |
| VEGF | TGCACCCACGACAGAAGGAGA | GCACTCCAGGGCTTCATCGTT |
| PI3K | ATGCACGGCGATTACACTCTTAC | ACTGGGTAGAGCAACTTCACATCC |
| PCNA | GAACCTCACCAGCATGTCCAAAAT | TTCACCCGACGGCATCTTTATTAC |
| P-Akt | AACAGAACGACCAAAGCCAAATAC | TGGGCTACAATTCATCCTCTCCT |
| FLK-1 | CTTCTGCAAAACACTCACCATTCC | GGATACCTAGCGCAAAGAGACACA |
| Id1 | CCTAGCTGTTCGCTGAAGGC | CTCCGACAGACCAAGTACCAC |
| Id2 | ATGAAAGCCTTCAGTCCGGTG | AGCAGACTCATCGGGTCGT |
| Id3 | TGTCGTCCAAGAGGCTAAGAG | TGCTACGAGGCGGTGTGCTG |
| Hes1 | GTTGTTTCACACGAGCCGTTCGCGTG | CACGCGAACGGCTCGTGTGAAA |
| Rap1G | CTCGCTCAAGTATGATGTCATCG | GGTAAAGCACGGGGTAGAATC |
| GAPDH | ACTTCAACAGCAACTCCCACTC | TAGGCCCCTCCTGTTATTATGG |

**Table S2. Summary of sequencing data quality.**

| **Sample** | **Raw_reads** | **Clean_reads** | **Error_rate** | **Q20** | **Q30** | **GC_pct** |
| --- | --- | --- | --- | --- | --- | --- |
| AST-1 | 85273304 | 82219056 | 0.03 | 97.05 | 92.22 | 49.9 |
| AST-2 | 110808524 | 106896100 | 0.03 | 96.79 | 91.69 | 49.08 |
| AST-3 | 94026028 | 91338756 | 0.03 | 96.75 | 91.61 | 49.95 |
| A-iSC-1 | 97377036 | 93264400 | 0.03 | 96.85 | 91.8 | 48.57 |
| A-iSC-2 | 109146708 | 104502320 | 0.03 | 96.85 | 91.83 | 49.17 |
| A-iSC-3 | 119500508 | 115504604 | 0.03 | 96.91 | 91.95 | 49.83 |
| P56-NPC-1 | 117588276 | 114277112 | 0.03 | 96.78 | 91.65 | 47.89 |
| P56-NPC-2 | 113922224 | 110587324 | 0.03 | 96.81 | 91.72 | 47.95 |
| P56-NPC-3 | 112925484 | 109801740 | 0.03 | 96.92 | 91.92 | 47.72 |
| P0-NPC-1 | 101428140 | 97288044 | 0.03 | 96.98 | 92.1 | 49.55 |
| P0-NPC-2 | 132385148 | 127027424 | 0.03 | 96.96 | 91.97 | 49.25 |
| P0-NPC-3 | 129547888 | 123757632 | 0.03 | 96.96 | 92.02 | 48.98 |
